# Effects of mid-gestational l-citrulline supplementation to twin-bearing ewes on umbilical blood flow, placental development, and lamb production traits

**DOI:** 10.1093/tas/txab102

**Published:** 2021-06-09

**Authors:** Michelle L Kott, Stefania Pancini, Savannah L Speckhart, Lauren N Kimble, Robin R White, Jamie L Stewart, Sally E Johnson, Alan D Ealy

**Affiliations:** 1 Department of Animal & Poultry Science, Virginia Polytechnic Institute and State University, Blacksburg, VA 24061, USA; 2 Department of Large Animal Clinical Science, Virginia Polytechnic Institute and State University, Blacksburg, VA 24061, USA

**Keywords:** citrulline, lamb performance, placenta, pregnancy, uterus

## Abstract

The objective of the study was to examine how l-citrulline supplementation to ewes during mid-gestation influences placental activity, placental blood flow, lamb body weight, and carcass characteristics. Two studies were completed. A pharmacokinetic study to compare circulating plasma amino acid concentrations after a single intravenous injection of 155 µmol/kg BW l-citrulline or after an isonitrogenous amount of l-alanine (control; 465 µmol/kg BW). Increases (*P* < 0.05) in circulating citrulline concentrations were detected for 8 h after l-citrulline injection versus the control. Similarly, increases (*P* < 0.05) in circulating arginine concentrations were detected for 24 h after l-citrulline treatment. The second study used 12 ewes with twin pregnancies. Daily intravenous injections of either l-citrulline or l-alanine were administered for 39 d from d 42–45 to 81–84 of gestation. Ewes were limit-fed at 85% daily energy requirements during the injection period. A decrease (*P* < 0.0001) in body weight was observed in both treatment groups during this period. No treatment differences were observed in circulating pregnancy-specific protein B concentrations or placental blood flow during the treatment and post-treatment gestational period. No treatment differences were observed in lamb survival nor in lamb birth, weaning and slaughter weights. Treatment did not influence lamb carcass composition or organ weights. However, there was a tendency (*P* = 0.10) for an increase in antral follicle numbers in ovaries from ewe lambs derived from ewes treated with l-citrulline. In summary, a daily l-citrulline injection increased both circulating citrulline and arginine concentrations in ewes, but daily l-citrulline injections during mid-gestation did not produce any detectable changes in placental activity and blood flow, neonatal and postnatal lamb development, and lamb carcass composition at slaughter. In conclusion, no benefits in placental function and lamb development were observed after providing l-citrulline during mid-gestation in ewes exposed to a mild energy restriction, but there was an indication that follicle numbers in ewe lambs were positively influenced by l-citrulline treatment during fetal development.

## INTRODUCTION

The maternal environment a fetus encounters during gestation can impact postnatal physiological function, growth potential, and lifetime productivity. This phenomenon is often referred to as developmental origins of health and disease or fetal programming (reviewed by Caton et al., 2020; Sartori et al., 2020). Gestational insults can influence fetal and postnatal development directly by affecting fetal organ and tissue development and indirectly by altering the epigenetic composition of chromatin (Reynolds and Caton, 2012). Various detrimental effects of these programming changes have been observed. For example, undernutrition during gestation in cattle and sheep compromises postnatal carcass composition, organ size, and onset of puberty in calves and lambs (Ford and Long, 2011; Long et al., 2012; Mwangi et al., 2019; Knight et al., 2020). However, there are circumstances where fetal programming events may be beneficial. For instance, increasing the plane of nutrition in beef cows during late gestation can improve daughter fertility (Martin et al., 2007; Cushman et al., 2014).

This work explored l-citrulline supplementation as a means for programming fetuses for improved postnatal lamb development and meat production. The non-essential amino acid, citrulline, is a precursor for arginine production, and both amino acids are metabolized to nitric oxide throughout the body, including in the placenta and uterus (Wu et al., 2013). Nitric oxide-dependent vasodilation and angiogenesis have been implicated in mediating early embryonic development, implantation, and placental perfusion rate in mammals presumably by providing more nutrients and oxygen to the uterus during pregnancy (Maul et al., 2003). Citrulline and arginine are also metabolized into ornithine and proline, which are substrates for polyamine synthesis (Wu et al., 2013). Polyamines are key players in embryonic and fetal gene expression, protein synthesis, and angiogenesis (Lenis et al., 2017). The reproductive benefits of supplementing these amino acids are best described in pigs, where dietary l-arginine supplementation to gestating sows and gilts improves placental growth, piglet birth weight, and embryonic survival (Mateo et al., 2007; Gao et al., 2012; Li et al., 2014). Increases in lamb birth weights have also been observed following l-arginine supplementation in ewes experiencing intrauterine growth restriction or in ewes that carried quadruplets (Lassala et al., 2010, 2011; Zhang et al., 2016a, 2016b; Peine et al., 2018). Moreover, increasing uterine blood flow with a vasodilator (Sildenafil citrate; Viagra) increases fetal size in sheep (Satterfield et al., 2010). For this study, l-citrulline was used in place of l-arginine because of citrulline’s greater half-life in circulation and its ability to sustain elevated circulating concentrations of arginine for longer periods of time than treating with l-arginine (Wu et al., 2007; Lassala et al., 2009).

The objective of the study was to examine the ability of l-citrulline administration during mid gestation to circumvent the negative effects of twin pregnancies and moderate energy restriction on placental activity, placental blood flow, lamb body weight (BW), and lamb carcass characteristics.

## MATERIALS AND METHODS

### Animal Use

All animal work was completed in compliance with and with the approval of the Virginia Tech Institutional Animal Care and Use Committee (Protocol #18–102). Suffolk × Dorset crossbred sheep used in this work were provided by the Virginia Tech Sheep Center (Blacksburg, VA).

### Treatment Preparation and Administration

l-citrulline (Bulk Supplements; Henderson, NV) was injected into the jugular vein at a concentration of 155 µmol/kg BW as this dosage generated a sustained increase in circulating citrulline concentrations in work by another laboratory (Lassala et al., 2009). l-alanine (Research Products International; Mount Prospect, IL) served as the control. Isonitrogenous administration required that 465 µmol/kg BW l-alanine be administered each day. Treatments were prepared within 2 h of their use in warmed USP grade sterile saline (0.9% [w/v] sodium chloride, Vet One, Boise, ID) to a final concentration of 0.13 g/mL l-citrulline and 0.11 g/mL l-alanine. Treatments were administered into the jugular vein after cleaning the neck with 70 % [v/v] ethanol. A topical analgesic and anti-inflammatory agent (Surpass^®^, Boehringer Ingelheim Vetmedica, Inc., St Joseph, MO) was applied as needed to limit hematoma formation. The location of injection sites varied each day, and different sides of the neck were used each week.

### Experimental Design

The first study was a pharmacokinetic pilot study. Mature, non-pregnant ewes (*n* = 3/treatment) received l-citrulline or l-alanine on a single day. Blood samples were taken immediately before and 0.5, 1, 2, 4, 8, and 24 h after treatment. Blood samples were maintained on ice until plasma was isolated via centrifugation (1500 × *g* for 15 min). Plasma was stored at −20 °C. Free amino acid concentrations were measured by using cation-exchange chromatography coupled with post-column ninhydrin derivatization and quantitation at the University of Missouri Agricultural Experiment Station Chemical Laboratories (Columbia, MO).

The second study utilized mature, twin-bearing ewes (*n* = 6 ewes/treatment). Ewes were selected from a larger group of ewes (*n* = 60 ewes) maintained on pasture at the Virginia Tech Sheep Center. Ewes received an Eazi-Breed CIDR sheep insert (0.3 g progesterone; Zoetis Inc., Kalamazoo, MI) for 14 d, and then were exposed to 2 Dorset × Suffolk and 2 Dorset rams for 4 d beginning 24 h after CIDR removal. Pregnancies were diagnosed by transabdominal b-mode ultrasonography on d 36–39 of gestation (d 0 = estimated day of breeding) using an ExaPad Mini ultrasound equipped with a 5-mHz sector probe (IMV Imaging, Rochester, MN). Twin-bearing ewes were assigned randomly to treatments after blocking based on age and BW. Beginning at d 38–41 of gestation (i.e., 2 d after ultrasonography), all 12 ewes were comingled in one dry-lot pen. Intravenous injections of l-citrulline or l-alanine occurred once daily at 0900 h for a 39 d period that spanned from d 42–45 to 81–84 of gestation. Ewe BW was recorded each week during the experimental period and approximately monthly thereafter until birth. One l-citrulline treatment ewe lost her pregnancy. None of the data collected from this ewe was included in the analyses.

Ewes were returned to normal farm management at the completion of the injection period. Ewes lambed indoors and transitioned to shelters containing pasture access after 7 d. Ram lambs were castrated within 1 wk of birth by castration banding, where an elastrator band was placed around the neck of scrotum ensuring that both testicles were below the band.

Lamb BW were taken at birth (d 0), d 7, 63–67 (weaning), 124–128 and 176–180 (1–5 d before slaughter). At weaning, 1 wether and 7 ewe lambs were derived from l-citrulline-treated ewes and 5 wethers and 4 ewe lambs were derived from l-alanine-treated ewes.

### Animal Diets and Feed Analyses

During the injection period, ewes were limit-fed to an estimated 85% of the NRC requirements for ME for gestating ewes (NRC, 2007) by providing 0.9 kg tall fescue hay/head/day provided over two feedings/day. All hay was consumed each day. Ewes had free access to water and trace mineral salts (Sweetlix Mineral with Bovatech, Sweetlix, Mankato, MN). Dietary intake of protein, vitamins, and minerals met or exceeded dietary requirements for mature, pregnant ewes. The amino acid composition of the diet during the treatment period is provided in Table 1. At the end of the treatment period, ewes were provided unobstructed access to Tall fescue pasture and were supplemented with 1 kg/head/day cracked corn in one group pen. The lamb pre-weaning diet consisted ad libitum access to creep feed. Post-weaned lambs were maintained on pasture. Biweekly samples were collected from hay, pasture, and grains. All feed samples were frozen, then thawed and dried in a forced-air oven for 72 h at 55 °C to determine DM. Samples were ground in a hammer-mill and passed through a 1-mm sieve, then a composite sample was produced for each time period and feed type and analyzed (Dairy One Forage Laboratory, Ithaca, NY). Nutrient analyses of all diets are provided in Table 2.

**Table 1.** Amino acid quantification in feed ingredients offered to ewes during the treatment period

Amino acid	g/100 g (as fed)
Hydroxyproline	0.03
Aspartic acid/asparagine	0.54
Threonine	0.27
Serine	0.23
Glutamic acid/glutamine	0.63
Proline	0.32
Lanthionine	0.00
Glycine	0.34
Alanine	0.38
Cysteine	0.08
Valine	0.36
Methionine	0.10
Isoleucine	0.27
Leucine	0.50
Tyrosine	0.16
Phenylalanine	0.33
Hydroxylysine	0.05
Ornithine	0.01
Lysine	0.25
Histidine	0.08
Arginine	0.26
Tryptophan Citrulline	0.04 ND

ND = not detected.

**Table 2.** Nutrient composition of diets offered to ewes and lambs throughout the study

		Ewe		Lamb	
	Treatment period	Post-treatment Pre-lambing		Pre-weaning	Post-weaning
Chemical composition	Hay	Hay	Cracked corn	Creep feed	Pasture
Dry matter, %	88.3	70.4	90.4	91.0	26.4
Crude protein, %	8.3	11.6	8.3	19.7	17.8
Neutral detergent fiber, %	73.9	68.9	7.0	11.4	57.1
Acid detergent fiber, %	47.6	43.7	2.5	5.6	32.6
Non-fiber carbohydrates, %	7.8	9.6	79.1	57.7	14.3
Metabolizable energy, Mcal/kg	1.92	2.02	3.44	3.39	2.38

### Doppler Ultrasonography

Transrectal color doppler ultrasonography was performed by a single technician 1 wk before the end of the treatment period (d 71–73) and 2 wk prior to expected lambing (d 134–137). Ewes were maintained upright and blood flow assessment was performed using an ExaPad Mini equipped with a 5-mHz sector probe. Pulsatility index (PI), resistance index (RI), and systolic/diastolic ratio (S/D) were recorded in the umbilical artery and in one placentome.

### Blood Sampling

Jugular venipuncture was completed weekly throughout the treatment period in pregnant ewes prior to treatment administration. Samples were maintained on ice until plasma was isolated via centrifugation (1500 × *g* × 15 min) and then stored at −20 °C. Plasma pregnancy-specific protein B (PSPB) concentrations were completed by BioTracking Inc. (Moscow, ID) (Thompson et al., 2013). The intra-assay CV was 4.8%.

### Lamb Carcass Data

Lambs were slaughtered at the Virginia Tech Meat Laboratory (Blacksburg, VA) at d 179–183. Carcasses were weighed prior to chilling and after 24 h at 2 °C. Backfat thickness was measured perpendicular to the longissimus dorsi, and body wall thickness was measured 12.5 cm from the midline between the 12th and 13th ribs. Quality grade, yield and leg conformation score were assigned according to USDA standards (USDA, 1992). Dressing percentage (DP) was calculated as a ratio of hot carcass weight (HCW) to live weight.

Visceral organs were harvested after removal from the carcass. Wet weights for each organ was recorded. Reproductive tracts were collected from the ewe lambs. Connective tissue was trimmed away, vagina was trimmed off just after the cervix, and the remaining tract including the ovaries was weighed. Two individuals who were blinded to treatment determined antral follicle count (AFC) by counting all visible follicles on each ovary.

### Statistical Analyses

All statistical analyses were performed using the SAS software package (version 9.4, SAS Institute Inc., Cary, NC). Ewe served as the experimental unit for the analysis of ewe BW, amino acid pharmacokinetic data, PSPB concentrations, and Doppler data using least-squares ANOVA with the general linear model (GLM) procedure. Lamb served as the experimental unit for carcass traits and organ weights using least-squares ANOVA with the GLM procedure. Organ weights were expressed relative to BW. The model used for these analyses included treatment and lamb sex, when appropriate. Lambs were considered independent of their twin.

Repeated measurement analysis was completed in the pilot study by using the Mixed procedure in SAS. Main effects included treatment, time and their interaction. Ewe(treatment) served as the error term for treatment. The mixed procedure also was used to complete repeated measurement analyses in the second study to examine ewe BW, lamb BW, and ewe PSPB concentrations. Main effects included treatment, day, lamb sex (when appropriate), and interactions. Either ewe(treatment) or lamb(treatment) served as the error term for treatment effects. Differences between individual means within day were examined by completing least squares ANOVA. Lamb survival analysis was performed with chi-squared analysis using the Genmod procedure. The statistical model used for this analysis included the main effect of treatment and sex.

All data is presented as least square means ± SEM. A *P* ≤ 0.05 was considered statistically significant and 0.05 > *P*-value ≤ 0.10 was considered a tendency.

## RESULTS

### Pharmacokinetic Pilot Study

**Figure 1.**
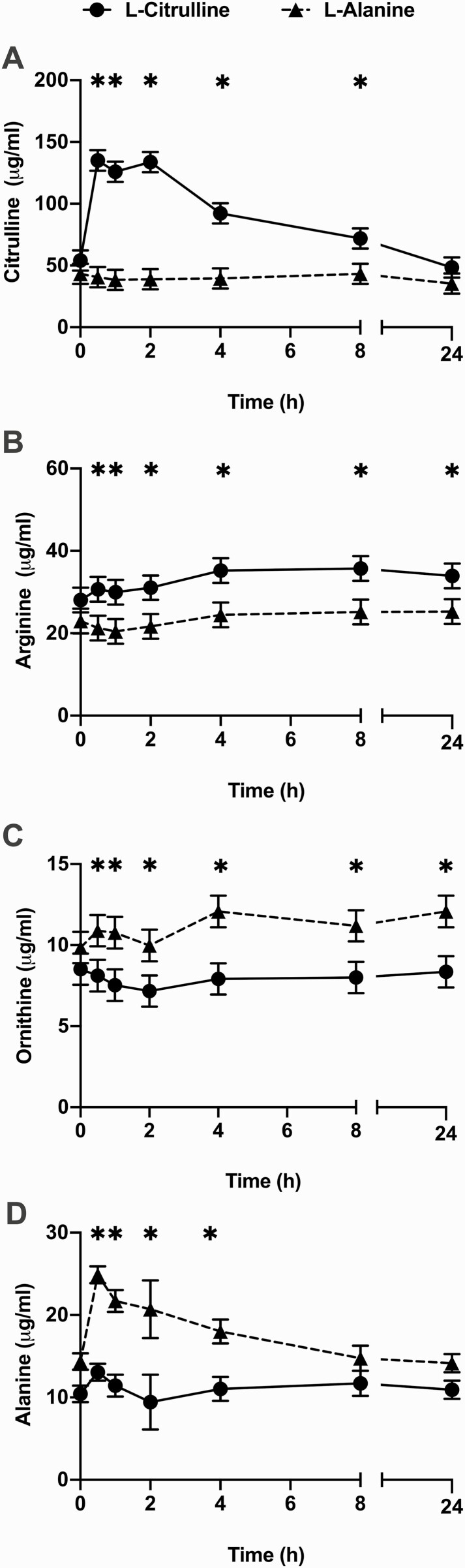
Circulating concentrations of selected AAs after a single intravenous bolus injection of l-citrulline or l-alanine to non-pregnant ewes. Blood samples were collected immediately before and 0.5, 1, 2, 4, 8, and 24 h after treatment (*n* = 3 ewes/group). l-citrulline supplemented ewes are indicated with a circle and solid line and l-alanine supplemented ewes are indicated with a rectangle and dashed line. Overall effects of l-citrulline were observed for circulating citrulline (A), arginine (B), and ornithine (C) concentrations (*P *< 0.05) whereas l-alanine injection only increased circulating alanine concentrations (D) (*P* = 0.02). Data represent least squares means and SEM. The asterisks (*) indicate differences between supplementation groups within each time point (*P* < 0.05).

Changes in circulating concentrations of citrulline, arginine, ornithine, and alanine after a single intravenous injection of l-citrulline or l-alanine (control) are presented in Figure 1. Ewes receiving an intravenous injection of l-citrulline exhibited an overall increase (*P *= 0.008) in circulating citrulline concentrations over a 24 h period. Increased (*P* < 0.05) citrulline concentrations were detected from 0.5 h to 8 h post-injection before returning back to control values at 24 h post-injection (Figure 1A). The intravenous l-citrulline injection also produced an overall increase (*P* = 0.003) in circulating arginine concentrations, where greater (*P* < 0.05) arginine concentrations were detected at each timepoint throughout the 24 h period (Figure 1B). The l-citrulline injection also caused an overall increase in circulating ornithine concentrations (*P* = 0.05), with greater (*P* < 0.05) ornithine concentrations detected at each timepoint during the 24 h period (Figure 1C). No changes in circulating concentrations of other free amino acids and urea were detected after l-citrulline injection (data not shown).

Injection of l-alanine did not influence citrulline or arginine concentrations but had an overall increase in circulating alanine concentrations (*P* = 0.02), with elevated alanine concentrations evident between 0.5 and 4 h but not thereafter (*P* < 0.05) (Figure 1D).

### Effects of Repeated l-Citrulline Injections on Ewe Parameters

**Figure 2.**
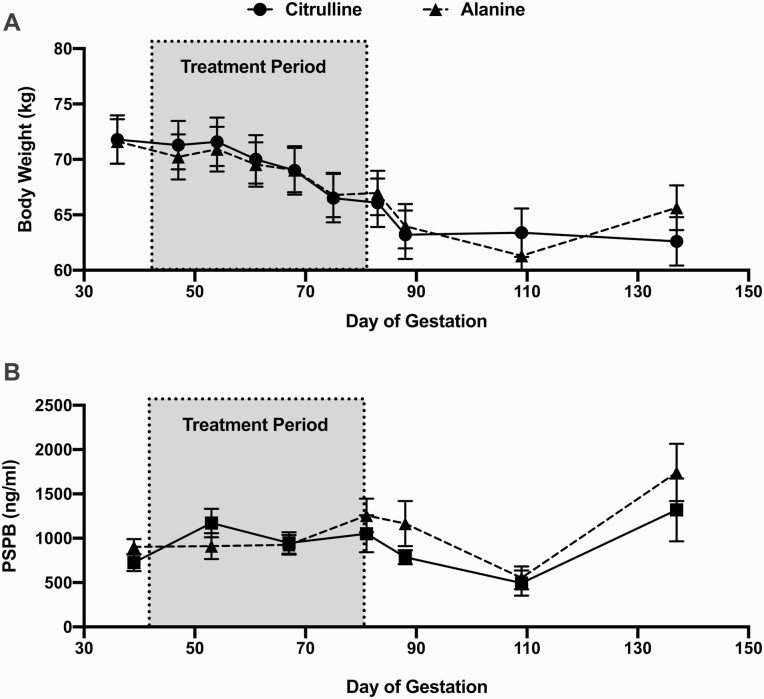
Ewe BW and circulating PSPB concentrations throughout and after the l-citrulline supplementation period. l-citrulline supplemented ewes are indicated with a circle and solid line and l-alanine supplemented ewes are indicated with a rectangle and dashed line. The greyed area indicates the treatment period. (A) BW data. (B) Circulating PSPB concentrations. Data represent least squares means and SEM.

At the beginning of the second study, initial ewe BW were 71.80 ± 2.20 and 71.62 ± 2.00 kg for l-citrulline and l-alanine injected ewes, respectively. Treatment did not affect BW but there was a reduction in BW in both groups over time (*P* < 0.0001). At the end of the 39 d injection period, BW had decreased by an average of 12.3% and 8.3% in the citrulline and alanine injection groups, respectively (Figure 2A). Circulating PSPB concentrations were unaffected by daily l-citrulline injections (Figure 2B). There was an overall effect of time on PSPB concentrations (*P* = 0.001). A time by treatment interaction was detected (*P* = 0.02) but PSPB concentrations were not different between treatments within each timepoint.

The final examination evaluated whether daily l-citrulline injections affected uterine and placental blood flow (Table 3). No effects of l-citrulline treatment were detected for RI, PI, and S/D ratio at d 71–73 and d 134–137 of gestation.

**Table 3.** Doppler ultrasonography results in pregnancies from ewes treated with l-citrulline during mid-gestation

	Umbilical vessel			Placentome		
	l-Alanine	l-Citrulline	*P*-value	l-Alanine	l-Citrulline	*P*-value
Day 71–73						
RI	0.59 ± 0.08	0.74 ± 0.09	0.22	0.43 ± 0.13	0.54 ± 0.15	0.61
PI	1.08 ± 0.15	1.39 ± 0.16	0.19	1.01 ± 0.45	0.97 ± 0.51	0.95
S/D ratio	4.04 ± 1.56	5.18 ± 1.71	0.64	2.07 ± 1.10	5.24 ± 1.42	0.13
Day 134–137						
RI	0.54 ± 0.06	0.64 ± 0.07	0.33	0.54 ± 0.08	0.53 ± 0.09	0.96
PI	0.87 ± 0.16	1.06 ± 0.18	0.44	0.90 ± 0.24	1.03 ± 0.29	0.74
S/D ratio	2.53 ± 0.48	3.15 ± 0.52	0.40	2.23 ± 0.85	3.52 ± 1.05	0.36

Values represent means in individual SEM.

RI = Resistance index, PI = pulsatile index, S/D ratio = systolic/diastolic ratio.

### Effects of Gestational l-Citrulline Treatment on Lamb Outcomes

**Figure 3.**
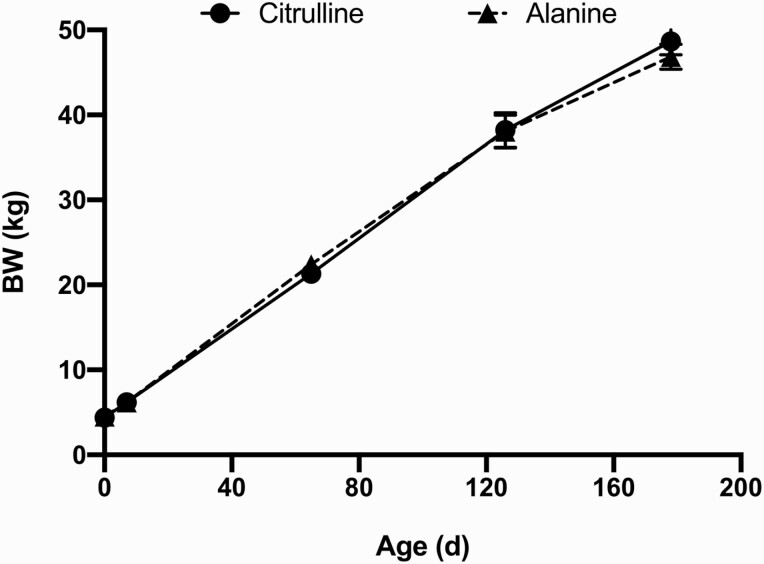
Body weights of lambs derived from ewes supplemented with l-citrulline during mid-gestation. Lamb BW were taken at birth, d 7, and approximately d 65, 126, and 178. l-citrulline supplemented ewes are indicated with a circle and solid line and l-alanine supplemented ewes are indicated with a rectangle and dashed line.

No differences in lamb survival were detected at birth and at weaning due to treatment, sex nor their interaction (Table 4). There was an effect of time (*P *< 0.0001) on lamb BW but neither treatment, treatment × time, nor sex influenced lamb BW (Figure 3). Within day analyses failed to identify any effects of treatment on lamb BW.

**Table 4.** Lambing outcomes from ewes treated with l-citrulline during mid-gestation

Parameter	l-Alanine	l-Citrulline	*P*-value
Sex distribution @ birth			
Ram lambs	7	2	-
Ewe lambs	5	8	-
Sex distribution @ weaning^1^			
Ram lambs	5	1	-
Ewe lambs	4	7	-
Carcass characteristics			
Live weight, kg*	47.11 ± 1.24	46.88 ± 1.53	0.91
Hot carcass weight, kg	23.07 ± 1.03	23.75 ± 1.27	0.69
Dressing percentage, %*	48.84 ± 1.29	50.95 ± 1.59	0.33
Backfat thickness, mm	3.48 ± 0.43	4.20 ± 0.53	0.32
Body wall thickness, mm	14.15 ± 1.51	17.54 ± 1.86	0.19
Adjusted fat thickness, mm	3.50 ± 0.54	4.71 ± 0.67	0.19
Loin muscle area, mm^2^	16.12 ± 0.51	16.40 ± 0.63	0.73
Leg score^2^	11.98 ± 0.23	12.23 ± 0.28	0.52
Quality grade^2^	10.76 ± 0.24	11.13 ± 0.30	0.35
Yield grade	1.78 ± 0.21	2.25 ± 0.26	0.19
(%BCTRC), %^3^	49.19 ± 0.36	48.58 ± 0.44	0.31

^1^Indicates lambs born alive that survived to weaning. Losses include stillbirths and postnatal losses.

^2^Scores/grade based on a numeric score of 10 = low choice, 11 = average choice, 12 = high choice.

^3^Percantage boneless closely trimmed retail cuts.

*Indicates sex-dependent differences (*P* ≤ 0.05).

The effects of l-citrulline supplementation on lamb carcass traits are presented in Table 4. No treatment and treatment by sex interactions were detected for any of the carcass traits. There was an effect of sex detected for dressing percentage, with wethers being heavier (*P* = 0.02) than ewe lambs (52.77 ± 1.72 % vs. 47.01 ± 1.19 %, respectively).

l-citrulline supplementation during gestation did not affect lamb heart, kidney, liver, lung, or pancreas weights at slaughter (Table 5). No treatment by sex interactions were observed, but main effects of sex were detected. Wethers had heavier (*P* = 0.04) pancreases and tended to have heavier (*P* = 0.09) lungs than ewe lambs (pancreas: 0.98 ± 0.10 vs. 0.68 ± 0.08 g/kg BW; lungs: 23.23 ± 2.53 vs. 17.29 ± 1.76 g/kg BW, for wethers and ewe lambs, respectively).

**Table 5.** Organ weights and other measurements in lambs derived from ewes treated with l-citrulline during mid-gestation

Parameter	l-Alanine	l-Citrulline	*P*-value
Organ wt. (g/kg BW)			
Heart	3.26 ± 0.15	3.30 ± 0.19	0.88
Kidney^1^	2.79 ± 0.10	2.76 ± 0.12	0.83
Liver	17.54 ± 0.65	18.64 ± 0.80	0.31
Lungs	20.96 ± 1.90	19.55 ± 2.34	0.65
Pancreas	0.79 ± 0.08	0.87 ± 0.10	0.55
Reproductive tract^2^	0.51 ± 0.06	0.52 ± 0.04	0.85
Ovaries			
Antral follicle count	30.00 ± 7.18	46.43 ± 5.42	0.10
Ewe lambs (*n*)	4	7	-

^1^Kidney weights were taken after perirenal fat was removed.

^2^Reproductive tract weights include the cervix, uterine horns, oviducts, and ovaries after connective tissue was trimmed.

In ewe lambs, overall reproductive tract weight was not influenced by l-citrulline supplementation, but there was a tendency (*P* = 0.10) for ewe lambs from citrulline-treated ewes to have greater AFC (Table 5).

## DISCUSSION

There are several reports in sheep indicating that supplementing l-arginine either intravenously or by feeding rumen-protected arginine can alleviate the severity of fetal growth restriction caused by underfeeding (Lassala et al., 2010, 2011; van der Linden et al., 2015; Zhang et al., 2016a, 2016b; Peine et al., 2018). This work utilized l-citrulline in place of l-arginine because of its greater half-life in circulation and its ability to sustain elevated circulating concentrations of arginine for longer periods of time than treating with l-arginine (Wu et al., 2007; Lassala et al., 2009). There is evidence that citrulline may escape rumen digestion after feeding (Gilbreath et al., 2020), and encapsulated, rumen protection schemes are available to deliver amino acids (de Chavez et al., 2015; Zhang et al., 2016b; Sun et al., 2017; Peine et al., 2018). However, we chose to provide l-citrulline directly into the circulation to ensure that sufficient amounts of citrulline were provided. Moreover, work by others suggested that intravenous l-citrulline supplementation is effective at sustaining elevated maternal plasma concentrations of both citrulline and arginine in sheep (Lassala et al., 2009). Our work also detected sustained increases in circulating arginine, citrulline, and ornithine concentrations, although the duration of elevated citrulline and ornithine concentrations differed from previous work, where citrulline was elevated for a longer period and ornithine for a shorter duration (Lassala et al., 2009). The same bolus administration scheme was followed in both in our study and the previous study, but ewes were not pregnant in this study whereas pregnant ewes were studied in the previous work. This potentially contributed to the slight alterations in circulating AA concentrations between these studies (Wu et al., 2007). Even so, the present pharmacokinetic study verified that the intravenous bolus treatment scheme increased arginine concentrations for the entire 24 h period and citrulline for at least 8 h. This allowed us to test the hypothesis that sustained increases in circulating arginine and citrulline concentrations benefit pregnancy in ways that will improve postnatal lamb development.

The gestational period when l-citrulline was supplemented (day 38–41 until day 81–84 of gestation) was chosen for two specific reasons. First, we wanted to wait until the first month of gestation was completed to avoid substantial early pregnancy losses and also to accurately detect twin pregnancies. Second, the termination of the study coincided with the stage of gestation when ewes normally would begin an energy supplementation regimen to ensure ewes possessed the body condition needed for late gestation and the post-partum periods. This interim window represented a time when pregnant ewes were normally maintained on stock-piled forages. This study focused on examining the consequences of limit-feeding a hay with low ME during this period of gestation. We were able to achieve body weight losses during the experimental period. This BW loss likely occurred because of the dietary restrictions, but we cannot rule out the possibility that low-level contaminants present in the amino acids contributed to this BW loss. Both amino acids met USP certification standards, but no purity testing was completed, so we can only speculate that there was little to no influence of contaminants on ewe BW and other outcomes.

Providing daily intravenous injections of l-citrulline did not alter any of the maternal parameters tested in this study. Reductions in BW were noted during the treatment period and l-citrulline did not alter the magnitude of BW loss, nor did it alter circulating PSPB concentrations, placental blood flow, and lamb birth weight. The lack of changes in PSPB concentrations suggest that l-citrulline did not alter placental function. Pregnancy specific protein B is a member of the pregnancy-associated glycoprotein (PAG) family (Telugu et al., 2009). These proteins are expressed solely by the placenta, and a subset of these PAGs, including PSPB, are released into the maternal circulation throughout pregnancy where they provide a useful blood-borne marker for pregnancy (Piechotta et al., 2011) and as an indicator of placental function in ruminants (Thompson et al., 2010; Wallace et al., 2015; Mercadante et al., 2016). One report detected improvements in overall cotyledon weight after l-arginine supplementation was observed at term, but this effect was not observed when pregnancies were terminated before term (van der Linden et al., 2015). Therefore, it remains unclear how influential l-arginine and l-citrulline are at mediating placenta development and function in ewes.

The umbilical cord and placentome are two of the more popular locations to examine blood flow (Morel et al., 2010; Vasquez-Hidalgo et al., 2020). There is one report describing benefits of rumen-protected l-arginine feeding on peripheral tissue blood perfusion rates (Peine et al., 2020), but we are not aware of any studies that directly quantified uterine or placental blood flow during l-arginine or l-citrulline supplementation in pregnant ewes. In fact, only a single report could be found that describes how cattle respond to intravenous l-arginine injections, and that study did not detect changes in uterine blood flow with l-arginine supplementation in pregnant dairy heifers (Yunta et al., 2015). Therefore, evidence from this work and others suggests that uterine and placental blood flow is unaffected by l-arginine or l-citrulline supplementation in ruminants.

This study also failed to detect changes in lamb birth weights after l-citrulline administration. This is consistent with what has been observed in non-restricted-fed ewes provided l-arginine through intravenous injection or feeding rumen-protected arginine (van der Linden et al., 2015; Crane et al., 2016; Gootwine et al., 2020). The work describing improvements in lamb birth weights following l-arginine supplementation have only been observed in ewes that underwent substantial feed restriction (50–60% of NRC recommendations for ME) (Lassala et al., 2010; Zhang et al., 2016a, 2016b; Peine et al., 2018) or in well-fed Booroola Rambouillet ewes heterozygous for the fecundity gene (FecB+/−) that were carrying quadruplets (Lassala et al., 2011). Thus, the apparent lack of improvements in placental function, placental blood flow, and lamb birth weight in this study likely occurred because the feed restriction was too mild (85% NRC requirements for energy) and ewes only carried twins. Additional nutritional and metabolic restrictions seem necessary before the benefits of l-arginine and potentially l-citrulline supplementation can be observed.

The final component of this study explored how gestational exposure to l-citrulline supplementation influenced postnatal lamb performance. This gestational treatment did not affect lamb BW. Most l-arginine studies did not evaluate lambs after birth, but interestingly, one recent report found that intravenous l-arginine injections for the first 2 weeks of pregnancy increased lamb weaning weight (Crane et al., 2016). More work is needed to explore how the timing of l-arginine or l-citrulline supplementation can impact lamb performance between birth and weaning.

Treatment with l-citrulline also failed to alter lamb carcass characteristics and organ weights. Primary myofibers emerge during embryonic development, and secondary myofibers develop and proliferate during mid and late gestation (Du et al., 2010). All muscle fiber hyperplasia ceases before birth, and postnatal muscle growth occurs through hypertrophy, not hyperplasia. Nutrient restriction of pregnant ewes during early embryogenesis causes a reduction in secondary fiber formation and a lower secondary:primary fiber ratio at mid-gestation in the fetuses that translates to a smaller muscle at birth with an altered metabolic profile (Zhu et al., 2004, 2006; Quigley et al., 2005). Manipulation of maternal diet also may be used to positively affect muscle fiber dynamics in the offspring. Nutrient restriction of beef cows during early gestation followed by realimentation of the dam to NRC recommendations causes compensatory growth of secondary fibers within the fetus (Gonzalez et al., 2013). Increased protein intake during gestation in cows also results in greater weaning weights and carcass weights at slaughter suggesting the macronutrient may be used to program efficient muscle deposition postnatally (Funston et al., 2010). Thus, there is a solid basis for proposing that maternal diet strategies are an effective means of altering offspring muscle content and composition. However, l-citrulline supplementation did not affect muscle content. We propose that the low energy deficiency of ewes in this study was not severe enough to cause muscle development problems.

Likewise, organ development was not affected by l-citrulline supplementation in this study. Supplementation with l-arginine can alleviate the adverse effects of severe energy restriction on organogenesis (Peine et al., 2018). Again, the low-level energy restriction in this study likely was not sufficient to detect any adverse effects on organs. Interestingly, there was a tendency for increased AFC was detected in ewe lambs derived from ewes given l-citrulline. This measurement was included in this study because AFC is linked with lifetime fertility in cattle (Ireland et al., 2008; Torres-Rovira et al., 2014; McNeel and Cushman, 2015; Pinto et al., 2018; Santa Cruz et al., 2018). Ovarian follicle reserves are established before birth. Therefore, it is exciting to speculate that exposing fetuses to l-arginine or l-citrulline will improve fertility in adulthood. However, more work is needed before any firm conclusions can be made about the linkage between fetal arginine and citrulline exposures and lifetime fertility potential.

To conclude, the injection scheme was effective at increasing circulating arginine and citrulline concentrations. Daily l-citrulline injections are not permissive for industry application, but this treatment scheme permitted us to ensure that we could detect any potential effects of l-citrulline treatment on fetal, placental and postnatal lamb development. There was no evidence supporting the hypothesis that mid-gestational l-citrulline supplementation to pregnant ewes fed will influence placental activity, placental blood flow, lamb birth weights, postnatal BW, carcass characteristics, and organ weights. We speculate that a more severe dietary restriction is needed to detect positive effects of l-arginine and l-citrulline on pregnancy and lamb performance parameters. Furthermore, greater animal numbers may be needed to adequately assess treatment effects for some parameters. Based on the variation noted in this work, increasing lamb numbers by 25–30% would be needed to provide adequate numbers for detecting l-citrulline effects on yield grade and doubling ewe lamb numbers would be needed to adequately assess how treatment influences antral follicle count. Increases Uncovering a potential benefit of l-citrulline supplementation on AFC offers new avenues of study aimed at describing how gestational exposure to arginine or citrulline could affect subsequent fertility of the offspring.
